# Sea shell diversity and rapidly evolving secretomes: insights into the evolution of biomineralization

**DOI:** 10.1186/s12983-016-0155-z

**Published:** 2016-06-07

**Authors:** Kevin M. Kocot, Felipe Aguilera, Carmel McDougall, Daniel J. Jackson, Bernard M. Degnan

**Affiliations:** School of Biological Sciences, University of Queensland, Brisbane, Queensland 4072 Australia; Current address: Department of Biological Sciences and Alabama Museum of Natural History, The University of Alabama, Tuscaloosa, Alabama 35487 USA; Current address: Sars International Centre for Marine Molecular Biology, University of Bergen, Thormøhlensgate 55, Bergen, 5008 Norway; Department of Geobiology, Goldschmidtstr.3, Georg-August University of Göttingen, 37077 Göttingen, Germany

**Keywords:** Biomineralization, Mollusc, Mantle, Shell, Shell matrix proteins, Co-option, Lineage-specific novelties, Repetitive low complexity domain

## Abstract

An external skeleton is an essential part of the body plan of many animals and is thought to be one of the key factors that enabled the great expansion in animal diversity and disparity during the Cambrian explosion. Molluscs are considered ideal to study the evolution of biomineralization because of their diversity of highly complex, robust and patterned shells. The molluscan shell forms externally at the interface of animal and environment, and involves controlled deposition of calcium carbonate within a framework of macromolecules that are secreted from the dorsal mantle epithelium. Despite its deep conservation within Mollusca, the mantle is capable of producing an incredible diversity of shell patterns, and macro- and micro-architectures. Here we review recent developments within the field of molluscan biomineralization, focusing on the genes expressed in the mantle that encode secreted proteins. The so-called mantle secretome appears to regulate shell deposition and patterning and in some cases becomes part of the shell matrix. Recent transcriptomic and proteomic studies have revealed marked differences in the mantle secretomes of even closely-related molluscs; these typically exceed expected differences based on characteristics of the external shell. All mantle secretomes surveyed to date include novel genes encoding lineage-restricted proteins and unique combinations of co-opted ancient genes. A surprisingly large proportion of both ancient and novel secreted proteins containing simple repetitive motifs or domains that are often modular in construction. These repetitive low complexity domains (RLCDs) appear to further promote the evolvability of the mantle secretome, resulting in domain shuffling, expansion and loss. RLCD families further evolve via slippage and other mechanisms associated with repetitive sequences. As analogous types of secreted proteins are expressed in biomineralizing tissues in other animals, insights into the evolution of the genes underlying molluscan shell formation may be applied more broadly to understanding the evolution of metazoan biomineralization.

## Background

According to the fossil record many animal phyla diversified during the Late Precambrian to Early Cambrian, roughly 515–541 million years ago (mya; [1, 2]). Various biotic and abiotic factors are hypothesized to have contributed to the rapid diversification of animal taxa at this time, including a three-fold increase in the concentration of calcium in seawater [1–7]. The dramatic increase in biomineralized skeletal structures over this period in multiple animal lineages is consistent with the convergent or parallel evolution of skeletogenesis in early animals.

**Fig. 1:**
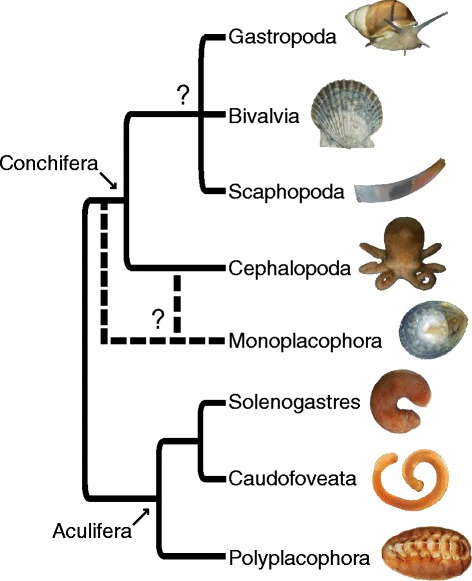
Current consensus of evolutionary relationships among the major lineages of Mollusca [10–12]. Photos are not to scale. Photo of *Argopecten* (Bivalvia) by Dan Speiser. Photo of *Chaetoderma* (Caudofoveata) by Christiane Todt. Photo of *Laevipilina* (Monoplacophora) by Greg Rouse and Nerida Wilson

Mollusca (snails, slugs, clams, squid, chitons and their allies) is one of the most morphologically and ecologically diverse metazoan phyla, with an estimated 200,000 extant species and an evolutionary history tracing back to at least to the Early Cambrian [5]. The great success of Mollusca can be attributed, at least in part, to their exoskeleton [5, 6], which provides defence and support. There are two major clades of Mollusca (Fig. 1; [7–9]): (i) Conchifera (Gastropoda, Bivalvia, Cephalopoda, Scaphopoda, and Monoplacophora), which includes all shell-bearing molluscs except chitons (Polyplacophora); and (ii) Aculifera, which includes Polyplacophora and the shell-less Aplacophora, a clade of molluscs that bear calcareous scales, spicules, or spines (collectively called sclerites) instead of one or more shells [10]. Although chitons have shells, their unique organization has prompted the hypothesis that chiton shells are not homologous to conchiferan shells ([10–13]; reviewed by [14]).

The adult molluscan shell is a remarkably stable organo-mineral biocomposite, in which the calcium carbonate mineral makes up 95–99 % [15]. In most molluscs, the outermost shell layer, known as the periostracum, is composed of organic components and is not calcified (but see [16]). The underlying shell layers primarily consist of aragonite and/or calcite polymorphs (rarely vaterite), and exhibit prismatic, nacreous, foliate, cross-lamellar or homogenous microstructures [13, 17, 18]. Little is known about the composition or microstructure of aculiferan sclerites.

### Diverse shell structures and patterns are produced from an homologous organ, the mantle

The initial formation of the molluscan shell occurs at the end of gastrulation, with the differentiation and local thickening of a group of ectodermal cells, which then invaginate into the blastocoel to form the shell gland [19, 20]. The shell gland evaginates to form the shell field, which then expands and differentiates into the mantle. Gene expression studies have revealed a number of conserved transcription factor and signalling ligand genes expressed in discrete zones within and around the developing shell field (e.g., [21–34], reviewed by [35]), suggesting that a deeply conserved gene regulatory network (GRN) lies at the heart of shell formation. The transcription factor *engrailed* is likely a key member of this GRN, as its expression has been observed at the boundary of non-shell-secreting and shell-secreting cells in the shell field margin of different molluscan classes [21, 22, 24, 28]. Gene knockdown of a second conserved developmental gene expressed in the shell field, the signalling ligand *decapentaplegic*, demonstrates that it operates downstream of *engrailed* and is required for the expression of shell-specific genes such as chitin synthase [34]. As the shell field is the precursor of the mantle, understanding the architecture of this larval shell-formation GRN and how it differs among the major lineages of Mollusca may be critical for elucidating the evolution of different shell morphologies and differences between shell versus sclerite-bearing taxa (e.g., Aplacophora).

**Fig. 2:**
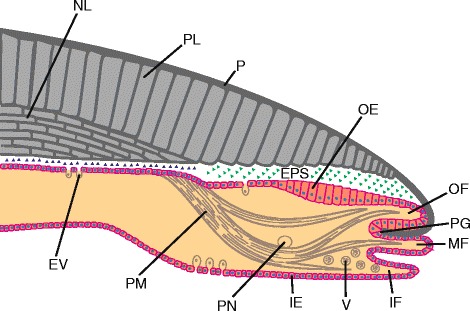
Schematic representation of a section through the shell and the mantle of a bivalve mollusc. Green and blue triangles depict organic macromolecules secreted by the mantle. NL: nacreous layer. PL: prismatic layer. P: periostracum. PG: periostracal groove. EPS: extrapallial space. OF: outer fold. MF: middle fold. IF: inner fold. OE: outer epithelium. IE: inner epithelium. PN: pallial nerve. PM: pallial muscle. V: vesicles. EV: exosome-like vesicles

The mantle of juvenile and adult conchiferan molluscs is divided into distinct morphogenetic regions consisting of highly specialized epithelial cell types [36–39] each responsible for the secretion of shell matrix macromolecules that influence the formation of specific shell layers. As an example, many bivalves and gastropods have a three-layered shell consisting of periostracum, prismatic, and nacreous layers; other shell constructions also occur in Gastropoda and Bivalvia. The outer periostracal layer is secreted from within a specialised groove found between the outer fold and remainder of the mantle (the periostracal groove; Fig. 2) [40, 41]. Production of the middle prismatic layer is controlled by genes expressed in columnar epithelial cells towards the extremity of the dorsal mantle surface, while production of the inner nacreous layer is controlled by genes expressed in cells in the inner zone of the mantle [42–45] (Fig. 2). Many of the genes expressed by these differentiated prism- and nacre-secreting mantle cells [46, 47] match with changes in shell features, such as structure, colouration and patterning [48–54], and have been identified and biochemically characterized with a wide range of potential functions including interacting with minerals, increasing shell strength, catalysis of enzymatic reactions, triggering of cell differentiation, stimulation of the synthesis of extracellular matrix components, and exertion of signalling activities towards the calcifying mantle epithelium (reviewed by [55–58]).

The dynamic spatial and temporal expression of shell-patterning genes (e.g., [41, 61]) demonstrate that regulation of shell biogenesis is complex, with different repertoires of developmental and structural genes being activated in different regions of the larval shell gland and juvenile/adult mantle at different stages of the life cycle. Given that it is the macromolecules secreted by the mantle (mantle secretome) that exercise control over shell morphology, one might expect that the diversity observed in shell structure is reflected by evolutionary changes in this mantle secretome, rather than changes in the master regulators acting within the mantle itself.

### The mantle secretome markedly differs between molluscs

In recent years, several studies have been conducted to identify proteins responsible for shell formation by isolating proteins contained in shells and/or genes specifically expressed in the mantle that encode a signal peptide, which indicate a protein is either secreted or localized on the cell surface. Studies employing transcriptomic approaches have significantly increased the number of identified and characterized genes expressed in the mantle of various bivalves [39, 60–76] and gastropods [59, 63, 77–79]. Although this method does not discriminate between genes involved in biomineralization and those that are not, *in silico* predictions of secreted proteins have been used to identify and compare putative shell matrix proteins (SMPs) in several taxa [63, 72, 78, 79]. Further, proteomic studies have revealed proteins secreted by the mantle that are actually incorporated into the shell [45, 64, 79–88], narrowing the gap in our understanding of gene expression in the mantle epithelium and the final destination of proteins in mineralized structures.

Despite advances in our understanding in this area, relatively few comparative studies have been performed and taxon sampling has been limited to just two of the eight of the major lineages of Mollusca (Bivalvia and Gastropoda). For example, Jackson et al. [63] compared the nacre-secreting mantle transcriptomes of a bivalve (*Pinctada maxima*) and a gastropod (*Haliotis asinina*), finding that the majority of the secreted proteins had no similarity to sequences in public databases, and less than 15 % of the secreted proteins were shared between the two species. These results indicate that the two taxa use different gene sets to construct their shells. This is in line with observations that both the crystallographic orientations of nacre tablets and their growth modes differ between these taxa, and strongly suggests that bivalve and gastropod mother-of-pearl nacre evolved convergently.

Similar results have been obtained at the proteome level. High levels of sequence novelty were found in the shell proteomes of the patellogastropod *Lottia gigantea* [83] and the heterobranch gastropod *Cepaea nemoralis* [79] when compared to public databases or to other gastropod and bivalve shell proteomes. Only 1.1 to 7.7 % of SMPs shared similarity between any two species; interestingly, the maximum level of similarity was found between a gastropod (*C. nemoralis*) and a bivalve (*Pinctada magaritifera*) [79].

Taken together, these studies indicate that SMPs directing shell formation in bivalves and gastropods, and even among lineages of gastropods, are markedly different. More closely related taxa do not necessarily have more similar SMP repertoires, indicating that the mantle secretome is extremely rapidly evolving. These genomic variations are likely to underlie the intra- and inter-specific differences observed in shell ultrastructure, shape, colour, pattern and strength.

### Ancient genes have been co-opted into shell formation

Although high levels of primary sequence novelty characterize most mantle transcriptomes and shell proteomes studied so far, the mantle also expresses genes with highly conserved domains including carbonic anhydrases, protease inhibitors, peroxidases, alkaline phosphatases and tyrosinases, among others ([37, 80, 89–107], Aguilera et al. unpublished data). These domains have roles outside of biomineralization and expression of genes encoding these domains is not limited to mantle tissue [72, 90, 93], suggesting that many SMPs have been co-opted independently in different molluscan lineages.

Phylogenetic analyses have revealed complex evolutionary histories for some of these co-opted domains. For instance, in many metazoan taxa, carbonic anhydrases (CAs) are characterized by multiple gene duplications coupled with independent co-options into a variety of skeleton-forming roles [92]. Similar to the evolutionary history of CAs, tyrosinase genes, at least in pearl oysters (*Pinctada* spp.) and the Pacific oyster (*Crassostrea gigas*), have expanded independently, with many of these duplicated genes being co-opted for mantle-specific functions [72, 93]. Likewise, dermatopontin genes have also undergone independent duplication and co-option events in the heterobranch gastropod clades Basommatophora and Stylommatophora [90]. Based on these observations and others [Aguilera et al. unpublished data], we propose that independent co-option and expansion of gene families are important driving forces acting on molluscan, and likely metazoan, biomineralization.

### Many proteins secreted by the mantle are encoded by rapidly evolving genes

As discussed above, mantle secretomes are composed largely of proteins with no sequence similarity to previously described molluscan biomineralization genes, as well as to publicly non-model mollusc databases. This degree of novelty poses challenges to orthology inference. Despite these difficulties, studies have addressed the evolution of lineage and species specific genes, with lysine (K)-rich mantle proteins (KRMPs) and shematrins the most well-investigated gene families in molluscan biomineralization [104].

These gene families have undergone extensive duplications and divergences in different lineages of pearl oysters. For example, the KRMP gene family has undergone independent expansions in different lineages of the genus *Pinctada*, leading to unique species-specific set of paralogs. By contrast, the shematrin gene family expanded before the speciation of these oysters, resulting in at least eight orthology groups that differ by the gain, loss, and shuffling of motifs [104]. The consistently high level of expression of these gene families in mantle tissue ([65, 66, 68, 92]; Aguilera et al. unpublished data) suggests that this rapidly-evolving component of the mantle secretome is also essential in shell formation. Whether this innate evolvability of the mantle secretome confers any selective advantage to molluscs, or whether it is simply a by-product of the types of proteins required for the architecture of the shell itself is not well understood.

### Shell matrix proteins often contain repetitive, low complexity domains

A particularly striking feature of molluscan SMPs is the preponderance of repetitive, low-complexity domains (RLCDs). Most repeats are short, with around 10 amino acids per repeat unit, although others have long repeated motifs on the order of 75–200 amino acids in length [106–108]. Approximately 30 % of the SMPs identified from *Lottia, Haliotis* and *Pinctada* contain such repeats [45, 73, 80].

Different functions have been attributed to different RLCD-containing proteins including binding to chitin, providing flexibility or rigid rod-like support, and binding calcium ions (when the repetitive motif is acidic) [108]. In many cases these RLCDs have biased amino acid compositions, usually with a high proportion of glycine and alanine residues (e.g., [91]), explaining why these amino acids were found to be highly abundant in earlier amino acid analyses of shell matrices (e.g., [109, 111–113]). This particular repetitive amino acid composition results in a disordered protein with a hydrogel-like structure, leading researchers to liken these SMPs to spider silk fibroins [112–117]. This presence of low complexity domains also suggests that due to the absence of standard proteolytic cleavage sites, high throughput proteomic methods now used to survey shell material are significantly less likely to detect these kinds of molecules.

Structural disorder of matrix proteins has generally been accepted as a feature of biomineralized structures in many taxa [118–120] and, interestingly, is associated with biased amino acid compositions and protein repetitiveness [120]. Therefore, the presence of RLCDs in biomineralization-associated proteins may reflect their tendency to adopt an intrinsically disordered conformation. Notably, a peptide derived from the molluscan biomineralization-associated protein pearlin/n16 is an important model for studying the behaviour of disordered proteins [121].

Interestingly, a survey of 39 molluscan aragonite-associated proteins revealed that all possessed a predicted disordered region [122], and it was hypothesised that this characteristic likely drives the assembly of the shell matrix in a process analogous to that which occurs in the vertebrate extracellular matrix [123]. Like the molluscan shell proteome, the human extracellular proteome is significantly enriched in proteins comprising more than 50 % of disorder compared to the complete human proteome. In molluscs, these proteins appear to function in promoting [124, 125] or inhibiting [125–127] crystallization of aragonite or calcite and modulating the morphology of the structures that are produced [122].

### Repetitive low complexity domains promote the rapid evolution of shell proteins

**Fig. 3:**
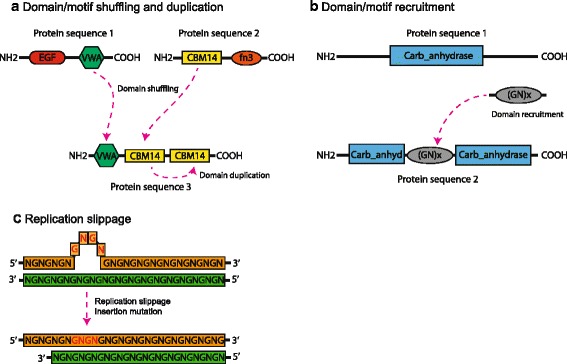
Potential evolutionary modes acting on SMPs. Three different evolutionary modes could explain the diversity of SMPs. **a** Domain/motif shuffling and duplication. **b** Domain/motif recruitment. **c** Replication slippage. This mode could produce the expansion or contraction of sequence repeats. In this case, the amino acids in red are inserted by replication slippage of codons

Many of the novel genes comprising the mantle secretome include RLCD-containing proteins [63, 78, 82]. These domains can either be in completely novel domain configurations or be combined with more ancient domains, such as observed in carbonic anhydrases [92]. Given the repetitive nature of the sequences encoding these domains, they may evolve through replication slippage and are susceptible to gain, loss and swapping of domains (Fig. 3). Considering that these repeats are often heterogeneous, other molecular mechanisms may also contribute to their origin, expansion and contraction.

Despite the unclear origin and evolution of RLCD-containing proteins, their prevalence suggests that proteins containing these domains are important components of the mantle secretome [63, 78, 82]. The apparent high rate of evolution of RLCD-containing proteins may contribute – at least in part – to the high levels of gene novelty found in all mantle secretomes examined to date. The molecular mechanisms underlying the evolution of molluscan shells is likely to be highly dynamic and characterized by independent gene family expansions, domain shuffling and co-option of genes. This variety of evolutionary modes acting on the terminal nodes of shell-forming processes may provide an explanation as to how an evolutionary homologous tissue can give rise to the great diversity of shell types seen in nature.

### Beyond molluscs: common principles in the evolution of skeletal proteins

A number of proteins and domains involved in biomineralization in molluscs appear to have similar functions in other animals [108]. For example, alpha carbonic anhydrase appears to be involved in biomineralization in diverse metazoans [92, 128–131]. Likewise, proteins with a C-type lectin domain are commonly associated with animal biomineralization [100–102, 108], including in a diverse repertoire of sea urchin skeletal matrix proteins [96, 97], the vertebrate pancreatic stone protein (lithostathine; [103]) and the avian eggshell protein ovocleidin 17 [132, 133]. The molluscan protein perlucin contains a C-type lectin domain and has a carbohydrate-binding ability thought to facilitate calcium-dependent glycoprotein-protein interactions within the skeletal matrix, which appears to promote the nucleation and growth of CaCO_3_ crystals [134]. Interestingly, deuterostome C-type lectin domain-containing skeletal matrix proteins do not have the carbohydrate-binding activity found in most C-type lectins [135]. Thus, despite their clear involvement in biomineralization, their exact function remains unclear.

Although other proteins that have roles in biomineralization in particular taxa have been shown to be conserved, their general role in biomineralization is less clear. For instance, MSP130, which is involved in biomineralization in the sea urchin (e.g., [96, 97]), is present in diverse metazoans including Annelida, Brachiopoda, Cephalochordata, Echinodermata, Entoprocta, Hemichordata, Mollusca, and possibly Porifera ([102, 103], Kocot unpublished data), including species that do not appear to produce mineralized structures (e.g. entoprocts).

As described above for molluscs, biomineralizing tissues in other animals express a high proportion of rapidly evolving gene families [136, 137]. For example, the biomineralizing proteomes of rhynchonelliform (articulate) brachiopods is comprised of a large number of novel, often acidic, proteins [126, 137, 138]. Other metazoan skeletal matrix protein repertoires also consist of a disproportionate number of acidic proteins, which directly interact with positively charged calcium ions triggering crystal nucleation [110] and affect polymorph selection and the growth of crystal step-edges [139]. Acidic proteins can also trigger the formation and stabilization of amorphous calcium carbonate [140, 141], which appears to be the initial phase of biomineralization in many animals (reviewed by [55, 142]). This is the current understanding of the roles of negatively charged proteins of calcium carbonate matrices but more work studying the binding affinity and capacity of these proteins is needed.

Finally, repetitive sequences similar to those found in molluscs are also common in skeletal matrix proteins in disparate metazoans. RLCD-containing proteins such as collagens, silks, and silk-like proteins are commonly observed in metazoan skeletal matrices, including in the echinoderms [143, 144], vertebrates [145, 146], arthropods [147] and brachiopods [126, 137, 138]. Different repeats have been hypothesized to have different roles but most appear to be involved in binding chitin or other macromolecules or in imparting flexibility or fracture resistance to the skeleton [108].

## Conclusions

The integration of the fields of genomics and proteomics into the study of molluscan biomineralization has revealed that shell formation is controlled by the highly coordinated expression of hundreds of genes, and the regulated secretion of proteins and other macromolecules. Although the dissection of the mantle gene regulatory network controlling shell fabrication is in its infancy, there is evidence, at least in early developmental stages, for a deep conservation of expression patterns of regulatory genes. Despite this apparent deep homology, the diverse array of molluscan shell architectures and patterns indicate that there exist underlying molecular differences that manifest later in the morphogenetic program. One source of this variation is the rapidly-evolving mantle secretome that shows high levels of uniqueness, even in closely related taxa. We propose that as terminal nodes in the mantle GRN, genes encoding the mantle secretome are less constrained and more evolvable, allowing for the intra- and inter-specific variation that underpins the spectacular diversity of molluscan shells.

Common principles that govern the molecular basis of skeleton formation are emerging from the analysis of molluscan SMP-encoding genes. These appear to apply broadly across the animal kingdom, and include (i) continuous influx and efflux of conserved secreted gene products, (ii) the evolution and expansion of lineage-specific secreted protein families, and (iii) the presence of highly-evolvable repetitive low complexity domains in both evolutionarily young and old secreted gene products. As in molluscs, these gene classes are likely to sit at the termini of late biomineralization GRNs in other animals. Further insight into how these ancient and novel gene families contribute to the building and patterning of the diversity of molluscan shells is likely to provide guiding principles into the evolution and formation of metazoan skeletons.

## Abbreviations

CA, carbonic anhydrase; GRN, gene regulatory network; KRMP, lysine (K)-rich mantle protein; RLCD, repetitive low-complexity domain; SMP, shell matrix protein
